# Photothermal Killing of A549 Cells and Autophagy Induction by Bismuth Selenide Particles

**DOI:** 10.3390/ma14123373

**Published:** 2021-06-18

**Authors:** Yue You, Jinxia Li, Linlin Chen, Mei Wang, Xinghua Dong, Liang Yan, Aiping Zhang, Feng Zhao

**Affiliations:** 1 CAS Key Laboratory for Biomedical Effects of Nanomaterials and Nanosafety, Institute of High Energy Physics, Chinese Academy of Sciences (CAS), Beijing 100049, China; youyue@ihep.ac.cn (Y.Y.); lijinxia@ihep.ac.cn (J.L.); chenll@ihep.ac.cn (L.C.); wangmei@ihep.ac.cn (M.W.); dongxh@ihep.ac.cn (X.D.); yanliang@ihep.ac.cn (L.Y.); 2 Department of Pharmaceutical Analysis, College of Pharmacy, Shanxi Medical University, Taiyuan 030001, China; zhangap1@163.com

**Keywords:** bismuth selenide, photothermal killing, apoptosis, autophagy, stress-related signaling pathway

## Abstract

With a highly efficient optical absorption capability, bismuth selenide (Bi_2_Se_3_) can be used as an outstanding photothermal agent for anti-tumor treatment and shows promise in the field of nanotechnology-based biomedicine. However, little research has been completed on the relevant mechanism underlying the photothermal killing effect of Bi_2_Se_3_. Herein, the photothermal effects of Bi_2_Se_3_ particles on A549 cells were explored with emphasis put on autophagy. First, we characterized the structure and physicochemical property of the synthesized Bi_2_Se_3_ and confirmed their excellent photothermal conversion efficiency (35.72%), photostability, biocompatibility and ability of photothermal killing on A549 cells. Enhanced autophagy was detected in Bi_2_Se_3_-exposed cells under an 808 nm laser. Consistently, an elevated expression ratio of microtubule-associated protein 1 light chain 3-II (LC3-II) to LC3-I, a marker of autophagy occurrence, was induced in Bi_2_Se_3_-exposed cells upon near infrared (NIR) irradiation. Meanwhile, the expression of cleaved-PARP was increased in the irradiated cells dependently on the exposure concentrations of Bi_2_Se_3_ particles. Pharmacological inhibition of autophagy by 3-methyladenine (3-MA) further strengthened the photothermal killing effect of Bi_2_Se_3_. Meanwhile, stress-related signaling pathways, including p38 and stress activated protein kinase/c-Jun N-terminal kinase (SAPK/JNK), were activated, coupled with the attenuated PI3K/Akt signaling. Our study finds that autophagy and the activation of stress-related signaling pathways are involved in the photothermal killing of cancerous cells by Bi_2_Se_3_, which provides a more understanding of photothermal materials.

## 1. Introduction

Nowadays, the development of strategies to completely cure cancer is still a great challenge. Novel technologies for fighting cancer are expected to be developed with enhanced efficiency, decreased toxicity and reversed drug multi-resistance. In recent years, photothermal therapy (PTT) has been gradually accepted for tumor therapy owning to its excellent effectiveness with minimal invasiveness and good compatibility [1,2]. It has also been used as an adjunct to pre-cancerous lesions and reduced residual tumor burden after operation [3,4]. It also can cooperate with traditional chemotherapy and radiotherapy in improving cancer treatment [5]. Under light irradiation, photothermal agents convert light energy into heat energy and increase the intratumoral temperature, leading to the death of cancer cells [6,7]. Thus, it is feasible to target the lesions directly, non-invasively to the surrounding healthy tissues.

This proves that the efficiency of PTT largely depends on the targeting capability of photothermal agents and their photothermal conversion efficiency [8]. The current research on photothermal therapy is focused on rapidly developing nanomaterials with great PTT potentials arising from the unique physiochemical properties of nanostructures, including a strong optical absorption, an enhanced photothermal conversation efficiency, a controllable surface multi-functionalization and the ability to deeply penetrate tissues [9]. Quite differently from the organic dyes with a lower near-infrared (NIR) light absorption coefficient and severe photobleaching, nanomaterials have shown good performance as photothermal agents in PTT [10]. Many nanomaterials have been reported to have excellent performances as photothermal agents. Among them, metal-containing nanoparticles have attracted great attention for photothermal conversion owing to their excellent photothermal conversion ability and good biocompatibility [11]. In recent years, bismuth-based nanomaterials have been introduced as attractive theranostic agents due to their extraordinary thermoelectric, photoelectric and optical properties [12,13]. Bismuth (Bi) has been used extensively as a medicine for its gastroprotective effects and also has great potential for medical bioimaging owning to its high atomic number [14,15]. Selenium (Se), one of the important trace elements in human, is present as the organ formations of selenocysteine and selenomethionine in the human body. It is essential for many enzymes’ activities and has also been reported in immunoregulation and cancer prevention. Bismuth-based nanomaterials, for instance, bismuth selenide, (Bi_2_Se_3_), has aroused intense interest among scientists. Importantly, Bi_2_Se_3_ has favorable biocompatibility, greatly facilitating their promising application. Bi_2_Se_3_ was reported to be degraded, and Se released from Bi_2_Se_3_ played an important role through selenoproteins to protect membranes and tissues [16], prevent cancers [17,18] and regulate immunity [19]. It was reported that Bi_2_Se_3_ nanoplates were intraperitoneally injected into mice at a high dose of 20 mg/kg and finally, 93% nanoplates were metabolized with few side-effects within the 90-day long-term period [20]. Based on the good safety profile of Bi_2_Se_3_ nanomaterials, the research on their potential applications has attracted great attention. Bi_2_Se_3_ has been used to develop promising theranostics platforms for cancer therapy by virtue of its good safety profile, excellent X-ray computerized tomographic (CT) imaging and photoacoustic imaging, coupled with photothermal and photodynamic therapeutic effects [21]. The ultra-thin Bi_2_Se_3_ nanoparticles synthesized by Xie et al. were reported to produce a significant tumor photothermal effect with a good photothermal conversion capacity [22]. Macrophage membrane-camouflaged hollow Bi_2_Se_3_ nanoparticles loaded with quercetin were found to increase photothermal sensitivity and potently inhibit lung metastasis of breast cancer [23]. All of these reports have revealed the satisfactory performances of Bi_2_Se_3_ in PTT. However, the molecular mechanism underlying the photothermal effect of Bi_2_Se_3_ nanomaterials remains elusive and needs further exploration.

Autophagy, a self-degradative system, plays a vital role in maintaining cellular homeostasis [24]. It is usually considered as a self-defense mechanism and defends cells from various environmental stimulation and cellular stresses such as heat, hypoxia, DNA damage, reactive oxygen species (ROS) and aggregation of misfolded proteins. Afterward, autophagy was found to be intimately implicated in cancer [25], and targeting autophagy has been regarded as a promising strategy for cancer treatment [26]. However, accumulating evidence has demonstrated that the effect of autophagy on cancer may be sophisticated and depends on tumor type, development stage and tumor microenvironment [27]. Heat stress has been recognized as a trigger for autophagy [28] and even the involvement of autophagy in the PTT effect has also been reported. Zhou and his colleagues found that autophagy inhibition could sensitize the hyperthermia-induced killing of cancer cells [29]. Afterward, they found that beclin-1-induced autophagy up-regulation might destroy the homeostatic functions of autophagy and activate autophagy death pathways, thus improving the efficacy of photothermal killing [30].

Autophagy may affect apoptosis dependently on the type and state of the cell. Autophagy can contribute to pro-survival pathways, while inappropriate autophagy can cause cell death [31,32]. Autophagy could be regulated by cell stress-related signal pathways, for instance, stress activated protein kinase/c-Jun N-terminal kinase (SAPK/JNK) [33] pathway and p38 MAP kinases (p38) pathway [34]. SAPK/JNK, a mitogen-activated protein kinases (MAPK) subfamily [35], generally induces apoptosis and growth inhibition in response to some stressors, including UV irradiation and oxidative stress [36]. Many data have indicated the associations between JNK signaling and cancer [37]. Actually, JNK has been considered as an attractive target for therapeutic intervention [38]. JNK activation can inhibit tumor formation and has a pro-apoptotic effect [39]. Similarly, p38 pathway is also activated responsively to various stresses and becomes involved in different cell processes, including autophagy [40] and cell death [41].

**Figure 1:**
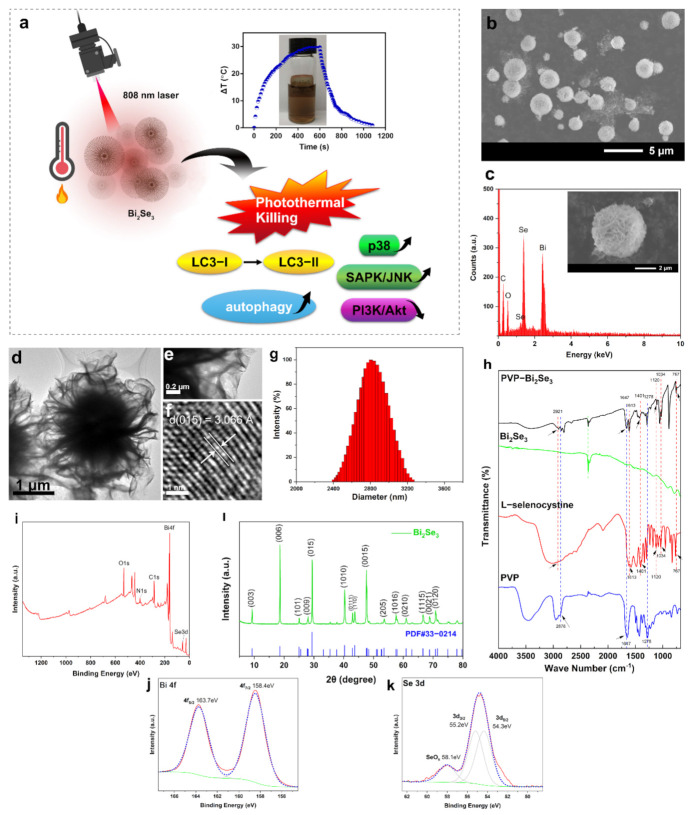
Physicochemical characterizations of Bi_2_Se_3_. (**a**) Scheme shows that the synthesized Bi_2_Se_3_ performed a remarkable photothermal killing of A549 cells with autophagy involved; (**b**) SEM image of PVP-Bi_2_Se_3_; (**c**) EDS spectrum of Bi_2_Se_3_. The insert shows a high-magnification SEM image; (**d**) TEM image of the synthesized Bi_2_Se_3_; (**e**,**f**) Magnified TEM image; (**g**) The size distribution of Bi_2_Se_3_ dispersed in water; (**h**) FTIR spectra of PVP-Bi_2_Se_3_, Bi_2_Se_3_, L-selenocysteine and PVP; (**i**) Survey scan XPS spectrum of PVP-Bi_2_Se_3_; HR-XPS spectra of (**j**) Bi 4f and (**k**) Se 3d scans of PVP-Bi_2_Se_3_. The solid lines represent the data curves, while the dotted lines are the fitted curves; (**l**) XRD patterns of PVP-Bi_2_Se_3_.

In this study, we intend to investigate the photothermal conversion capability as well as the photothermal killing effect of the synthesized Bi_2_Se_3_ particles. Emphasis may be put on the exploration of the underlying biological mechanisms, including autophagy and the stress-related signaling pathways (Figure 1a).

## 2. Materials and Methods

Dimethyl sulfoxide (DMSO), RPMI-1640 culture medium and phosphate buffer solution (PBS) were purchased from Hyclone (Hyclone Laboratories, Logan, UT, USA). Fetal bovine serum (FBS) and penicillin-streptomycin solution were purchased from Gibco (Gibco Invitrogen, Grand Island, NY, USA). Cell-counting kit-8 (CCK-8), Annexin V-FITC/PI assay kit and calcein-AM/PI double stain kit were purchased from Dojindo (Dojindo Laboratories, Tokyo, Japan). All primary antibodies (LC3β, cleaved-PARP, p-p38, p-SAPK/JNK, p-Akt and GAPDH) and secondary antibodies were purchased from Cell Signaling Technology (Cell Signaling Technology, Danvers, MA, USA). ECL prime Western blot detection reagent was purchased from GE Healthcare (GE Healthcare UK Limited, Little Chalfont, Buckinghamshire, UK). All aqueous solutions were prepared from a Milli-Q water system (Merck Millipore, Billerica, MA, USA). A549 human lung adenocarcinoma cell line and human umbilical vein endothelial cells (HUVEC) were obtained from American Type Culture Collection (ATCC).

### 2.1. Characterization of Bi_2_Se_3_ Particles

Bi_2_Se_3_ particles were synthesized as described in the previous work of our group. In brief, to synthesize Bi_2_Se_3_ particles, 1.0 g of poly (vinylpyrrolidone) (PVP) dissolved in 19.5 mL of deionized water was heated to 80 °C in a water bath under argon protection. Then, 10 mL of L-selenocysteine dissolved in deionized water (3 mM) and 0.6 mL of NaOH (0.5 M) were slowly added to the flask and the sample was maintained at 80 °C for 10 min. Afterward, 0.5 mL of Bi (NO_3_)_3_ solution (0.1 M) was quickly added to the flask while vigorously stirring. The obtained solution was irradiated with visible light and kept at 80 °C for 3 h. During this period, the color of the solution gradually changed to brownish-black, indicating the formation of the product. As the reaction ended, the mixture was stood to cool to room temperature. The final product was washed three times with deionized water, dialyzed to further remove impurities and finally stored at −20 °C for use. Then, the morphology, size and microstructures of synthesized Bi_2_Se_3_ were determined by a field emission scanning electron microscope (SEM, Hitachi S-4800, Tokyo, Japan) and a transmission electron microscope (TEM, Tecnai G2 F20 U-TWIN, Hillsboro, OR, USA). The elemental compositions of the particles were determined with an energy-dispersive spectrometer (EDS) attached to SEM. Dynamic light scattering (DLS) analyzer (NanoBrook Omni) (Brookhaven Instruments, Holtsville, State NY, USA) was used to measure the hydrodynamic diameter and zeta potential of Bi_2_Se_3_ dispersion in purified water. Fourier Transform Infrared (FTIR) spectrometer (Thermo Scientific, Waltham, MA, USA) with Nicolet iN10 MX spectrograph (Thermo Scientific, Waltham, MA, USA) was used to record the formation of PVP-Bi_2_Se_3_ in the powder form. To detect chemical structures of the formation of Bi_2_Se_3_, X-ray photoelectron spectroscopy (XPS, Thermo Scientific K-alpha, Thermo Scientific, Waltham, MA, USA) measurements were performed to characterize the chemical stoichiometry with monochromatic Al Kα radiation (1486.6 eV). X-ray diffraction (XRD) patterns of the sample were performed by a Bruker D8 Advance X-ray diffractometer. The measurements were operated in the refection mode with Cu Kα radiation, and the 2θ range between 5° and 80° were recorded.

### 2.2. Photothermal Conversion Performance of Bi_2_Se_3_ Particles

UV-visible-near-infrared (UV-Vis-NIR) absorption spectra of Bi_2_Se_3_ with different concentrations (100, 200 μg/mL) were collected by UV-Vis spectrophotometer (UV 1800, Shimadzu Scientific Instruments, Shimadzu, Japan) with a wavelength coverage of 300–1000 nm. To further detect the photoabsorption capability, the extinction coefficient ε (λ) of the Bi_2_Se_3_ was calculated. Various concentrations of Bi_2_Se_3_ dispersions (0, 25, 50, 100 μg/mL) were irradiated with 808 nm laser (VLSM-808-B, CONNET FIBER OPTICS, Shanghai, China) (1.0 W) for 10 min. Calibration and determination of the laser source were performed using a Thorlabs optical power meter (PM100D, THORLABS GmbH, Dachau, Germany) (0.93W), and temperatures were monitored by an infrared thermal imaging instrument (FLIR i7, FLIR Systems, Wilsonville, OR, USA). The photostability test was performed by irradiating Bi_2_Se_3_ dispersion with an 808 nm laser for 10 min and then turning off the laser for four cycles.

### 2.3. CCK-8 Cell Viability Assay

A549 and HUVEC cells were cultivated in RPMI-1640 medium supplemented with 10% fetal bovine serum (FBS), 1% penicillin/streptomycin at 37 °C in a 5% CO_2_ and humid atmosphere, respectively. The medium is changed every 2 days until cells are 80% confluence.

To explore the cytotoxicity and photothermal killing effect of Bi_2_Se_3_, A549 and HUVEC cells were seeded into 96-well plates (1 × 10^4^ cells/well) and cultured overnight in a 37 °C incubator, respectively. Then, cells were incubated with a fresh medium containing different concentrations of Bi_2_Se_3_ (0, 6.25, 12.5, 25, 50, 100, 200 µg/mL) in the absence or presence of laser irradiation. Irradiation was performed using an 808 nm laser with an intensity of 0.21 W/cm^2^ and then cells were cultured for a further 12 h. Cell viability was tested using CCK-8 assay. Absorbance was measured in each well at 450 nm using a microplate reader (Victor X3, Perkin Elmer, Waltham, MA, USA).

### 2.4. Live and Dead Assay

Calcein-acetoxymethyl ester (Calcein-AM)/propidium iodide (PI) staining was a routine method for assessing the status of cells based on membrane integrity. Calcein-AM and propidium iodide solutions can stain live and dead cells, respectively. Structurally, the high lipophilicity of methyl acetate helps calcein-AM readily penetrate live cells. AM group can be removed by active esterase in live cells; thus, calcein emits strong green fluorescence. PI can only cross cell membrane that has lost its integrity, where it embeds in DNA double helix and produces red fluorescence. PI stains necrotic or late apoptotic populations, but not early apoptotic cell populations [42].

The well-grown cells were incubated with different concentrations of Bi_2_Se_3_ (0, 25, 50, 100, 200 µg/mL) in the absence or presence of laser irradiation. After 10 min of irradiation, cells were further cultured for 12 h and the treatment period ended. For calcein-AM/PI staining, cells were incubated with 2 μM calcein-AM and 2.5 μg/mL PI for 10 min at 37 °C. Representative images were obtained using a fluorescence-inverted microscope system (Olympus IX81, Olympus, Tokyo, Japan).

### 2.5. Annexin V-FITC/PI Double Staining

Annexin V-FITC/PI assay kit was used to evaluate the photothermal killing effect of Bi_2_Se_3_. Well-grown cells were incubated with Bi_2_Se_3_ (0, 25, 50 µg/mL) and subject to laser irradiation for 10 min followed by a further incubation of 12 h. Then, all the cells (both the attached and floating cells) were collected by trypsinization and centrifugation. The obtained cells were washed twice using phosphate buffered saline (PBS) solution, resuspended in 500 μL of binding buffer containing 5 μL of annexin V-FITC and 5 μL of PI, then incubated for 15 min in the dark at room temperature. Apoptosis was immediately analyzed by flow cytometry (Accuri C6, USA). After positioning the quadrants on the Annexin V-FITC/PI dot plots, live cells (Annexin V−/PI−), early apoptotic cells (Annexin+/PI−), late apoptotic cells (Annexin V+/PI+) and necrotic cells (Annexin V−/PI+) were distinguished.

### 2.6. Monodansylcadaverine (MDC) Staining for Autophagy Assay

A549 cells were seeded on confocal dishes (1 × 10^4^ cells/mL). After 24 h, cells were incubated with different concentrations of Bi_2_Se_3_ dispersions (0, 25, 50 µg/mL) for 6 h in the absence or presence of laser irradiation. At the end of exposure, cells were stained with 50 μM MDC for 20 min and Hoechst 33,342 dye for 5 min in the dark. Then, samples were loaded onto a laser confocal microscope and the representative images were obtained.

### 2.7. Western Blotting Analysis

Western blotting analysis was performed as follows for total proteins. At the end of exposure, A549 cells were washed with ice-cold PBS solution and lysed in RIPA lysis buffer supplemented with complete protease inhibitor cocktails (Roche, Basel, Switzerland) on ice. The solutions of cytolysis were centrifuged (12,000 rpm) for 15 min at 4 °C and the supernatant liquor was collected into cold tubes. The total protein content of each sample was determined using a bicinchoninic acid assay (BCA) protein detection kit (Applygen, Beijing, China). A unit of 20 μg protein was loaded and separated on a 12% sodium dodecyl sulfate-polyacrylamide gel electrophoresis (SDS-PAGE) and transferred onto 0.22 μm polyvinylidene difluoride (PVDF) membrane. Immediately, PVDF membrane was washed three times with Tris-buffered saline Tween-20 (1 × TBST) and then blocked in 5% bovine serum albumin (BSA) for 1 h at room temperature. The membrane was incubated with the indicated primary antibodies, including LC3β (1/3000), cleaved-PARP (1/4000), p-p38 (1/4000), p-SAPK/JNK (1/4000) and p-Akt (1/4000), overnight at 4 °C. PVDF membranes were washed three times with 1 × TBST and incubated with the corresponding peroxidase-conjugated secondary antibodies at room temperature for 1 h followed by three washes. Finally, target proteins were detected after incubation with an electrochemiluminescence (ECL) reagent and immunoreactive bands were captured using a chemiluminescence imaging system (Azure C300, Azure Biosystems, Dublin, CA, USA). GAPDH was used as an internal reference. Band intensity on the exposed film was semi-quantified using ImageJ software (National Institutes of Health, Bethesda, MD, USA). All experiments were repeated at least three times independently.

### 2.8. Statistical Analysis

All experiments in the current study were independently repeated at least 3 times with similar results. The relative percentage and values were presented as the Mean ± Standard Deviation (SD) of six parallel samples. Statistical analysis was performed by two-tailed Student’s *t*-test for unpaired data, with *p* < 0.05 considered statistically significant.

## 3. Results and Discussions

### 3.1. Characterization of Bi_2_Se_3_

Bi_2_Se_3_ was synthesized as described in *Methods and Materials*. Figure 1b shows the SEM images of Bi_2_Se_3_. The morphology of the synthesized Bi_2_Se_3_ was observed to be flower-like and spherical in shape with a size of ~2.90 μm. Figure 1c shows the corresponding EDS results of the Bi_2_Se_3_ sample, confirming the presence of Se and Bi. Typical transmission electron microscopy (TEM) images showed that the synthesized PVP-Bi_2_Se_3_ exhibited a relatively uniform structure with an average diameter of ~2.75 μm (Figure 1d). The high-resolution TEM (HRTEM) image presented in Figure 1e,f demonstrated clear lattice fringes with 3.066 Å spacing, corresponding to a lattice spacing of the (015) facets. The results from dynamic light scattering (DLS) analysis revealed that the average hydrated particle size of Bi_2_Se_3_ was approximately 2804.13 nm with good dispersion stability (Figure 1g), and their zeta potential was tested to be −32.61 mV. The characteristic peaks of L-selenocysteine included a broad band at 2921 cm^−1^ which is assigned to the amino group stretching vibration, and the bands at 1401 and 1613 cm^−1^ corresponding to the symmetric and asymmetric flexural vibration of the carboxylic group, respectively (Figure 1h). Besides, the stretching bands of C–N were at 1120 and 1034 cm^−1^, and the stretching band of C–Se was at 767 cm^−1^. The above absorption bands were found in the spectrum of our synthesized Bi_2_Se_3_, revealing the presence of L-selenocysteine on the surface of naked Bi_2_Se_3_. Besides, in the pure PVP spectrum, a very intense peak at 1647 cm^−1^ was due to the carbonyl stretching of the five-membered cyclic lactam structure. There were peaks at 2953 and 2876 cm^−1^ of spectrum related to C–H stretching for aliphatic compounds [43,44], of which the latter was found in the PVP- Bi_2_Se_3_. The band near 1278 cm^−1^ in the pure PVP spectrum due to ring C–N stretching coupled with ring CH_2_ wagging [45], which could be observed in the PVP- Bi_2_Se_3_ sample FTIR spectrum. This proved that PVP was coated on the surface of Bi_2_Se_3_. The typical XPS survey scan spectrum of synthesized Bi_2_Se_3_ shows the presence of Bi, Se and N elements in the sample (Figure 1i). Figure 1j,k shows the high-resolution XPS (HR-XPS) spectra of Bi 4f and Se 3d, respectively. As the Bi 4f spectrum shows (Figure 1j), the binding energies at 158.4 and 163.7 eV are assigned to Bi 4f_7/2_ and Bi 4f_5/2_, respectively. The peaks at 54.3 and 55.2 eV are corresponding to Se 3d_5/2_ and Se 3d_3/2_, respectively, which are consistent with the reported XPS data of Bi_2_Se_3_ [46,47,48]. Figure 1k also showed shoulders at 58.1 eV, which resulted from the formation of Se–O bonds, suggesting the oxidation of Bi_2_Se_3_ [47,49], and the oxidation has been reported to be commonly present in Bi_2_Se_3_ [49]. XPS results further confirmed the synthesized Bi_2_Se_3_, and the Bi and Se atoms of Bi_2_Se_3_ are in the valence state of −2 and +3, respectively. Figure 1l shows XRD patterns of Bi_2_Se_3_ determine a very strong (015) orientation peak, which is consistent with the plane orientation of Bi_2_Se_3_ in the HRTEM result (see Figure 1f). The strong diffraction peaks can be indexed as the layered rhombohedral phase of Bi_2_Se_3_ (JCPDS Card No. 33-0214). The well-defined peaks verified the formation of Bi_2_Se_3_ with high quality.

### 3.2. Photothermal Profile of Bi_2_Se_3_

**Figure 2:**
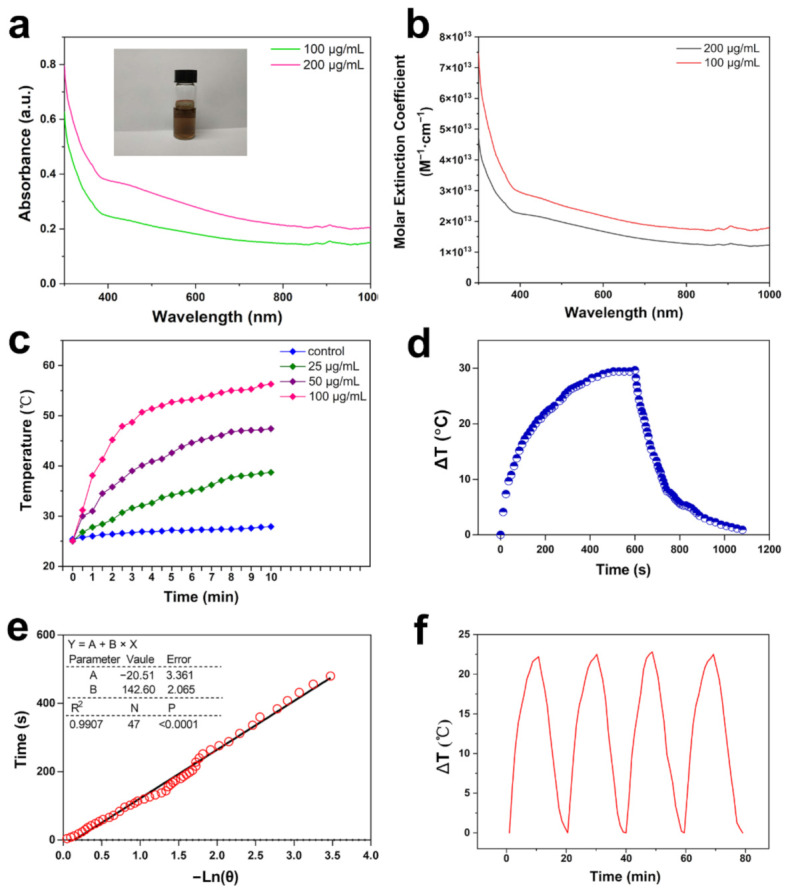
Photothermal profile of Bi_2_Se_3_. (**a**) Room-temperature UV–Vis-NIR absorbance spectra for the Bi_2_Se_3_ dispersed in deionized water (100, 200 μg/mL). The Insertedphoto shows 100 μg/mL Bi_2_Se_3_ dispersion; (**b**) the extinction coefficient $\left(\varepsilon \right)$ of Bi_2_Se_3_ particles (100, 200 μg/mL); (**c**) Temperature elevation curve of Bi_2_Se_3_ dispersion under 808 nm laser (0.21 W/cm^2^); (**d**) Photothermal effect of Bi_2_Se_3_ dispersion (100 μg/mL) with laser irradiation (808 nm, 0.21 W/cm^2^). The laser was removed after irradiation for 600 s; (**e**) The calculation of the heat conversion efficiency (η) of Bi_2_Se_3_ dispersions (100 μg/mL); (**f**) Photostability test curve of Bi_2_Se_3_ (50 μg/mL) within four laser on/off cycles (0.21 W/cm^2^).

Bi_2_Se_3_ powder was dispersed in water and the obtained dispersion was brown-black optically. The UV-visible-NIR absorption spectra of Bi_2_Se_3_ dispersion displayed a certain absorption in the NIR region, suggesting its potential photothermal efficacy (Figure 2a). The molar extinction coefficient $\varepsilon_{808}$ of the Bi_2_Se_3_ was calculated from the measured absorbance in Equation (1) [50,51]:

(1) $$\begin{array}{c} \varepsilon_{808}=\left(A_{808}V\rho N_{A}\right)/\left(LC\right) \end{array}$$

where *A* is the absorbance of the Bi_2_Se_3_at 808 nm wavelength (Figure 2a), *V* (unit: cm^3^) is the average volume of individual Bi_2_Se_3_ and *ρ* is the density of the Bi_2_Se_3_ (7.51 g/cm^3^) [52]. *N_A_* is Avogadro’s constant, *L* is the path length (unit: cm) and *C* (unit: g/L) is the weight concentration of the Bi_2_Se_3_ dispersion. The result demonstrated that the Bi_2_Se_3_ particles had a high ε with ∼1.7 × 10^13^ M^−1^·cm^−1^ at 808 nm (100 μg/mL) (Figure 2b). Besides, to directly evaluate the photothermal conversion capacity of Bi_2_Se_3_ particles, their thermal performance was monitored during laser irradiation of 10 min (Figure 2c). The temperatures of Bi_2_Se_3_ dispersions were tested to increase time- and concentration-dependently under laser irradiation and finally reached a platform, quite differently from the subtle change in the temperature of pure water upon irradiation. The temperature of 100 μg/mL Bi_2_Se_3_ dispersion sharply increased to 56.3 °C under laser irradiation (0.93 W), and the temperature increased to 47.4 °C of 50 μg/mL Bi_2_Se_3_ dispersion, indicating that the synthesized Bi_2_Se_3_ can efficiently convert 808 nm NIR energy into heat energy. To obtain the heat conversion efficiency (η) of Bi_2_Se_3_ dispersions (100 μg/mL), we recorded the temperature difference (ΔT) under the 808 nm laser. Until the temperature stopped rising, the irradiation source was shut off. The decline of the temperature was monitored (Figure 2d). According to the obtained data, we plotted the linear time data against negative values of the natural logarithm of the driving force temperature obtained from the cooling period (after 600 s) (Figure 2e). The time constant for the heat transfer was calculated to be $\tau_{s}$ = 142.6 s. Then, $\eta_{808}$ was calculated by Equation (2) [29,51,53]:

(2) $$\begin{array}{c} \eta_{808}=\frac{hS∆T_{max}-Qs}{I\left(1-10^{-A_{808}}\right)} \end{array}$$

where $\Delta T_{max}$ is the maximum stable-state temperature (29.67 °C) and *Qs* is the baseline energy input of deionized water, which is determined independently to be 0.005 mW. *I* is the laser power, 0.93 W. $\;A_{808}$ is the absorbance at 808 nm (0.15, Figure 2a) and  hS is obtained via Equation (3) [53].

(3) $$\begin{array}{c} hS=\frac{mC}{\tau_{s}} \end{array}$$

where *m* is the mass (g) of deionized water and *C* is heat capacity under constant pressure (J/g·°C). Substituting *hS* into Equation (2), $\eta_{808}$ of Bi_2_Se_3_ can reach ~31.13%. This result exhibited a remarkable photothermal conversion capacity possessed by the synthesized Bi_2_Se_3_. Then, we chose 50 μg/mL Bi_2_Se_3_ dispersion for the photostability test. We irradiated Bi_2_Se_3_ dispersion for 10 min for four cycles, separated by 10 min closure of irradiation to allow the temperature recovery, during which the alterations in temperature were carefully monitored. The results demonstrated that the photothermal conversion capacity was still maintained after four continuous heating and cooling cycles of Bi_2_Se_3_ particles, suggesting a good photostability (Figure 2f). Herein, the physicochemical properties of Bi_2_Se_3_ suggested it promising as a prominent photothermal agent.

### 3.3. The Photothermal Effect of Bi_2_Se_3_

**Figure 3:**
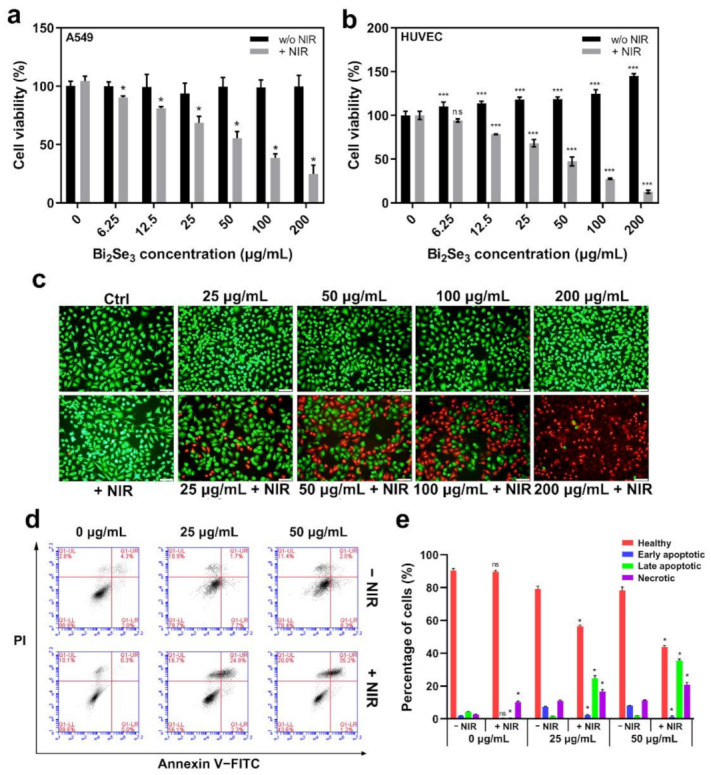
Photothermal killing of A549 cells by Bi_2_Se_3_ particles. The viability of A549 cells (**a**) and HUVEC cells (**b**) upon Bi_2_Se_3_ exposure (0, 6.25, 12.5, 25, 50, 100, 200 μg/mL) with or without laser irradiation. * *p* < 0.05, *** *p* < 0.01, versus untreated control group; (**c**) Live–dead staining of A549 cells. A549 cells were incubated with Bi_2_Se_3_ with or without laser irradiation. Cells with green fluorescence (calcein-positive cells) represent live cells while red fluorescence dead cells (PI-positive cells). Scale bar: 40 μm. The demonstrated images are representative of three independent experiments; (**d**) Apoptosis analysis by flow cytometry using Annexin V-FITC/PI staining kit. Cells were incubated with Bi_2_Se_3_ (0, 25, 50 µg/mL) and subjected to laser irradiation for 10 min followed by a further incubation of 12 h. Then, cells were harvested for apoptosis analysis with annexin V-FITC/PI staining. Representative flow cytometry data were presented; (**e**) The percentages of healthy, early apoptotic, late apoptotic and necrotic apoptotic cells in each group from the results of three independent experiments were quantified (* *p* < 0.05, versus without NIR group).

It is crucial to assess the cytotoxicity of Bi_2_Se_3_ when considering the potential applications in the biomedical field. Herein, A549 and HUVEC cells were incubated with various concentrations of Bi_2_Se_3_ dispersions ranging from 0 to 200 μg/mL, and cell viability was tested using a CCK-8 assay (Dojindo Laboratories, Tokyo, Japan) after 24 h. The results demonstrated that Bi_2_Se_3_ was nontoxic to both A549 and HUVEC cells, even at concentrations up to 200 μg/mL (Figure 3a,b). To investigate the photothermal killing of cancer cells by Bi_2_Se_3_ particles, Bi_2_Se_3_-incubated A549 cells were irradiated with an 808 nm laser for 10 min and then incubated for a further 12 h without irradiation. The results from the cell viability analysis demonstrated that under laser irradiation, Bi_2_Se_3_ particles induced a concentration-dependent decrease in the viability of A549 cells. Under laser irradiation, the viability of cells incubated with 12.5 μg/mL Bi_2_Se_3_ dispersion was approximately 80% of the control cells, while the cell viability decreased to 25% as the concentration of Bi_2_Se_3_ increased to 200 μg/mL (Figure 3a). Instead, neither only laser irradiation nor only Bi_2_Se_3_ exposure decreased cell viability. Herein, it showed that both laser irradiation and Bi_2_Se_3_ particles were required for the killing of cancer cells, suggesting a photothermal killing of cancer cells by Bi_2_Se_3_ particles. Meanwhile, the photothermal effect of Bi_2_Se_3_ on HUVEC cells was also tested (Figure 3b). The results revealed that upon laser irradiation, HUVEC cells treated with Bi_2_Se_3_ were damaged at concentrations above 12.5 μg/mL. Under 808 nm laser irradiation (0.21 W/cm^2^), the viability of cells incubated with 12.5 μg/mL Bi_2_Se_3_ dispersion was ~75% of the control cells, while the cell viability decreased to ~13% as the concentration of Bi_2_Se_3_ increased to 200 μg/mL. This implies that normal cells are probably more sensitive to the photothermal killing of Bi_2_Se_3_ than tumor cells. Considering its potential application, local interventional therapy exploiting PTT effect of Bi_2_Se_3_ is advised to minimize the off-target phototoxicity.

Calcein-AM/PI live–dead staining further confirmed the photothermal killing of Bi_2_Se_3_. It showed that only green fluorescence existed in the cells incubated with either Bi_2_Se_3_ or irradiation, indicating that no cytotoxicity was induced by only laser irradiation or only Bi_2_Se_3_. In Bi_2_Se_3_-exposed cells, upon NIR irradiation, red fluorescence appeared, revealing the photothermal cell death induced by Bi_2_Se_3_. Moreover, with laser irradiation, more red fluorescent spots were observed in cells incubated with 200 μg/mL Bi_2_Se_3_ dispersion than those incubated with 100 μg/mL dispersion, exhibiting a concentration-dependent photothermal killing of Bi_2_Se_3_ (Figure 3c). This result was highly consistent with the result from CCK-8 assay, together demonstrating a potent photothermal killing ability of Bi_2_Se_3_ particles advantageous for cancer therapy.

The photothermal killing of A549 cells by Bi_2_Se_3_ was further confirmed using an Annexin V-FITC/PI double-labeling kit (Dojindo Laboratories, Tokyo, Japan). The results from the flow cytometry analysis demonstrated that the apoptosis and necrosis rate of Bi_2_Se_3_-exposed cells combined with laser irradiation was significantly higher than that of the irradiation-only group or Bi_2_Se_3_-only exposure group. The death-inducing effect of Bi_2_Se_3_ exposure plus laser irradiation was also shown to be concentration-dependent (Figure 3d). The percentages of healthy, early apoptotic, late apoptotic and necrotic cells in each group were quantified in Figure 3e.

### 3.4. Autophagy Gets Involved in the Photothermal Killing of Bi_2_Se_3_

**Figure 4:**
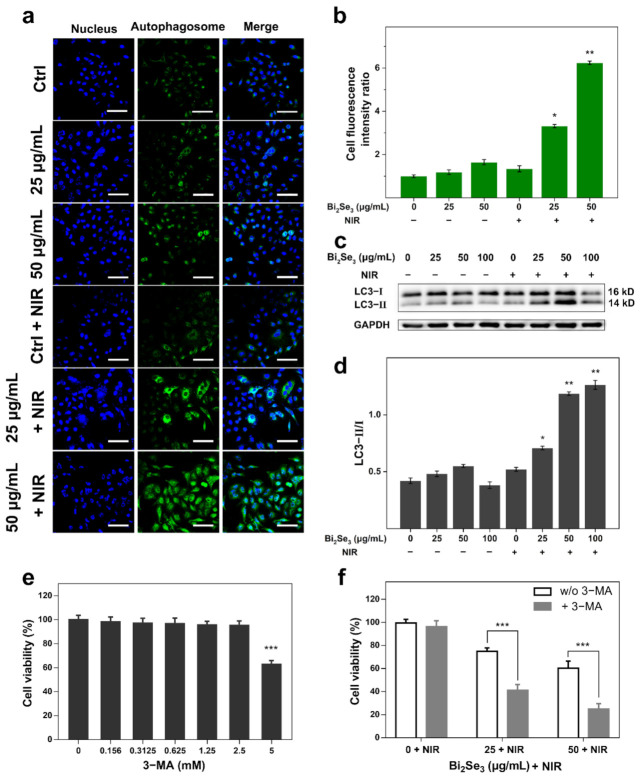
Autophagy induced by Bi_2_Se_3_ upon laser irradiation. (**a**) Autophagosome formation in Bi_2_Se_3_-incubated cells upon irradiation as observed by MDC staining. A549 cells were treated with diverse concentrations of Bi_2_Se_3_ (0, 25, 50 μg/mL) with or without laser irradiation. The nucleus was stained with Hoechst 33,342 (Beyotime Institute of Biotechnology, Shanghai, China) (blue) and autophagic vacuole was stained with MDC (green). Representative images were obtained using confocal fluorescence microscopy. Scale bar: 80 μm; (**b**) The mean fluorescence intensity of MDC dye in each group of A549 cells of 10 different fields was quantified using ImageJ. * *p* < 0.05, ** *p* < 0.01 versus control group (irradiated cells without Bi_2_Se_3_ incubation); (**c**) The increased LC3 II/I level in Bi_2_Se_3_-exposed cells upon laser irradiation. Cells were exposed to various concentrations of Bi_2_Se_3_ (0, 25, 50, 100 μg/mL) with or without irradiation. GAPDH serves as a loading protein. The representative data of three independent experiments are shown here; (**d**) The relative expressions of LC3 II/I in each group from the results of three independent experiments were quantified using ImageJ (* *p* < 0.05, ** *p* < 0.01); (**e**) The effect of 3-methyladenine (3-MA) on the basal cell viability of A549 cells; (**f**) The effect of 3-MA on the photodynamic killing of Bi_2_Se_3_. The data presented are representative of three independent experiments (*** *p* < 0.001).

In advance to high-temperature-induced cell death, autophagy is probably triggered to cope with harsh environments and cellular stress [29]. To explore whether autophagy was involved in the photothermal killing effect of Bi_2_Se_3_, autophagosome formation was observed first using Monodansylcadaverine (MDC) staining (Solarbio, Beijing, China). The results demonstrated that autophagy was induced in Bi_2_Se_3_-incubated cells upon laser irradiation. The intracellular bright green fluorescence illustrated the formation of autophagosomes in the cytoplasm of A549 cells [54]. slight green fluorescence was present in the control cells with no incubation of Bi_2_Se_3_. Laser irradiation itself also failed to trigger autophagy, as no obvious green fluorescence was observed in the cytoplasm of the irradiated cells without Bi_2_Se_3_ incubation. Additionally, no obvious autophagy was induced in Bi_2_Se_3_-incubated cells with no irradiation. Distinctly, a significant increase in the fluorescence intensity, representing autophagosome formation, was observed in the Bi_2_Se_3_-incubated cells upon laser irradiation, demonstrating autophagy induction by the photothermal role of Bi_2_Se_3_ (Figure 4a). Furthermore, upon laser irradiation, a higher concentration of Bi_2_Se_3_ exposure triggered more potent autophagy in cells. Then, the green fluorescence intensity in each group of A549 cells was quantified by ImageJ software (Version 1.8.0, 2021, National Institutes of Health, Bethesda, MD, USA) and presented in Figure 4b.

When autophagy occurs, partial cellular components are encapsulated in autophagosomes and eventually degraded in fusion with lysosomes. Microtubule-associated protein 1 light chain 3 (LC3) is a signature protein of autophagy, composed of cytoplasmic LC3 (LC3-I) and LC3-II spotted on the membrane of autophagosomes [29]. The conversion of LC3 protein from LC3-I to LC3-II is widely recognized as an indicator of autophagy behavior [55]. Autophagy induced by the photothermal effect of Bi_2_Se_3_ was also confirmed by an increased ratio of LC3-II/LC3-I. The results from western blotting analysis demonstrated that the abundance of LC3-II was increased obviously in Bi_2_Se_3_-exposed cells upon laser irradiation, especially in the group of 50 μg/mL dispersion (Figure 4c). It should be pointed out that the total expression of LC3 protein (both LC3-I and LC3-II) in the irradiated cells incubated with 100 μg/mL dispersion displayed a lower level, which was inferred to be related to the accelerated autophagy protein degradation at the high concentration. Compared with other groups, the ratio of LC3-II/I in Bi_2_Se_3_-exposed cells combined with laser irradiation was significantly higher (Figure 4d), indicating an enhanced autophagy level. Therefore, it confirmed that Bi_2_Se_3_ could induce autophagy in A549 cells under laser irradiation.

Then, we investigated the effect of 3-methyladenine (3-MA), a specific autophagy inhibitor, on the photothermal killing of Bi_2_Se_3_ in vitro. 3-MA, with concentrations of less than 2.5 mM, has marginal effects on the viability of A549 cells (Figure 4e). Notably, 1 h pretreatment of 1 mM 3-MA obviously increased the photothermal killing capability of Bi_2_Se_3_, as seen from the further decrease in cell viability by 3-MA pretreatment (Figure 4f). The results suggested that autophagy inhibition might enable a more efficient photothermal killing of Bi_2_Se_3_ particles.

To summarize, autophagy was enhanced in A549 cells by Bi_2_Se_3_ particles upon laser irradiation, and autophagy inhibition by 3-MA probably enabled a more efficient photothermal killing of Bi_2_Se_3_.

### 3.5. Activated Stress-Related p38 and SAPK/JNK Signaling Pathways Coupled with the Attenuated PI3K/Akt Signaling in the Photothermal Effect of Bi_2_Se_3_

**Figure 5:**
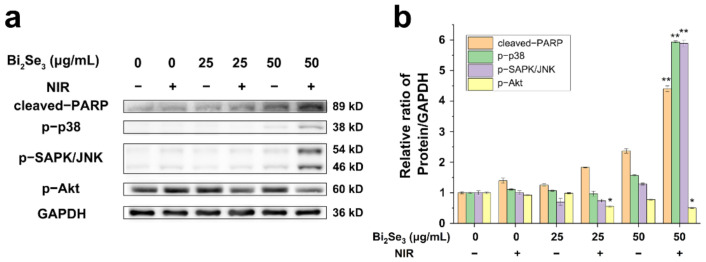
Effects of Bi_2_Se_3_ on intracellular stress-related and survival-associated signaling pathways. (**a**) The expressions of cleaved-PARP, phosphorylated p38 (p-p38), phosphorylated SAPK/JNK (p-SAPK/JNK) and phosphorylated Akt (p-Akt) in A549 cells under different conditions by western blot analysis. GAPDH serves as a loading protein. The representative data of three independent experiments are shown here; (**b**) The bands of these proteins of interest proteins from the results of three independent experiments were quantified using ImageJ. * *p* < 0.05, ** *p* < 0.01 versus control group. The data presented are representative of three independent experiments.

As demonstrated above, upon laser irradiation, Bi_2_Se_3_ induced a photothermal killing of cancer cells. In response to the hyperthermal stimuli, the intracellular stress-related signaling pathways, including p38 and p-SAPK/JNK are usually initiated. Using Western blot analysis, the phosphorylation levels of p38 and p-SAPK/JNK were detected to be increased in Bi_2_Se_3_-incubated cells upon irradiation. Especially in the irradiated cells incubated with 50 μg/mL Bi_2_Se_3_ particles, the phosphorylation levels of p38 and p-SAPK/JNK were dramatically increased, suggesting potent activations of p38 and p-SAPK/JNK signaling pathways. Accordingly, the expression level of cleaved-PARP, an apoptotic marker, was elevated obviously in the cells -incubated with 50 μg/mL Bi_2_Se_3_ upon irradiation (Figure 5a), consistently with the results from CCK-8 and live–dead staining. As expected, PI3K/Akt signaling, which is generally associated with cell survival and growth [56], was significantly attenuated in Bi_2_Se_3_-exposed cells upon irradiation. The phosphorylation level of Akt in Bi_2_Se_3_-exposed cells combined with irradiation was significantly reduced (Figure 5a). The results were quantified by ImageJ and demonstrated in Figure 5b with the increased levels of cleaved-PARP, phosphorylated p38 and phosphorylated SAPK/JNK, as well as the decreased level of phosphorylated Akt in Bi_2_Se_3_-incubated cells upon laser irradiation. Herein, the activated stress-related SAPK/JNK and p38 signaling pathways, coupled with the attenuated PI3K/Akt signaling, were involved in the photothermal killing effect of Bi_2_Se_3_.

## 4. Conclusions

In the present work, the synthesized Bi_2_Se_3_ exhibited good biocompatibility, excellent photothermal conversion capability and photostability. With laser irradiation, Bi_2_Se_3_ performed a significant photothermal killing of A549 cells via apoptosis mechanism. Moreover, autophagy induction was involved in the photothermal effect of Bi_2_Se_3_, which may be a self-protective behavior against the hyperthermal stimuli, as inferred from a more severe cell death triggered by pretreatment of 3-MA. Autophagy inhibition by 3-MA probably enabled a more efficient photothermal therapy. Simultaneously, stress-related p38 and p-SAPK/JNK signaling pathways were obviously activated, accompanied by the attenuated PI3K/Akt signaling. Our work provides new insight into the mechanisms underlying the photothermal effects of Bi_2_Se_3_. The effect of the material-mediated PTT is determined by many external parameters, such as the localization of the materials in the tumor, accumulation of materials within the tumor and interaction between materials and cell-membrane components. The research of these aspects deserves further exploration. Meanwhile, given the potential application, the explorations on the strategy for improving the tumor-targeting performance of Bi_2_Se_3_ particles as well as the ADME (absorption, distribution, metabolism and excretion) profile need to be conducted. We also intend to combine the photothermal effect with other therapies to further improve the therapeutic potential of Bi_2_Se_3_ for tumor eradication in vivo.

## Data Availability

Data sharing is not applicable to this article.
